# Long-term care needs and the risk of household poverty across Europe: a comparative secondary data study

**DOI:** 10.1186/s12877-024-04687-x

**Published:** 2024-01-26

**Authors:** Georgia Casanova, Roberto Lillini, Carolina Moreno, Giovanni Lamura

**Affiliations:** 1 Instituto de Investigación en Políticas de Bienestar Social (POLIBIENESTAR) - Research Institute on Social Welfare Policy, Universitat de València, Valencia, 46022 Spain; 2 Centre for Socio-Economic Research on Ageing, IRCCS-INRCA National Institute of Health & Science on Ageing, Ancona, 60124 Italy; 3 Data Science Unit, Department of Epidemiology and Data Science, Fondazione IRCCS Istituto Nazionale dei Tumori, Via Giacomo Venezian 1, Milan, 20133 Italy

**Keywords:** ADL disability, Long-term care, Ageing population, Risk of household poverty, Health and social public policies

## Abstract

**Purpose:**

Population ageing and rising poverty are two of the most pressing issues today, even in Western European nations, growing as a result of the recent global economic crisis and the COVID-19 containment measures. This study explores the relationship between long-term care (LTC) needs and risk of poverty at household level in eight European countries, representing the different European care regimes.

**Methods:**

The main international databases were scoured for study variables, categorized according to the following conceptual areas: home care, residential care, health expenditure, service coverage, cash benefits, private services, population, family, education, employment, poverty, disability and care recipients, and life expectancy. We initially identified 104 variables regarding 8 different countries (Austria, Finland, Germany, the Netherlands, Italy, Spain, Poland, Romania). Statistical analyses were conducted as described hereafter: analysis of the Pearson’s Bivariate Correlation between the dependent variable and all other variables; a Multivariable Linear Regression Model between the Poverty Index (dependent variable) and the covariates identified in the preceding step; a check for geographical clustering effects and a reduced Multivariable Linear Regression Model for each identified European cluster.

**Results:**

The variables that addressed the risk of poverty pertained to the area of policy intervention and service provision. Rising private out-of-pocket health expenditures and proportion of “poor” couples with at least one child are two factors that contributed significantly to poverty increasing. Moreover, rising private out-of-pocket health expenditures for covering LTC needs (even in presence of public financial contribution to the family) is the main contributor to household poverty increasing in presence of ADL disability.

**Conclusion:**

The results reveal the existence of a clear correlation between the need for LTC and the risk of poverty in households across Europe. These results highlight the central relevance of LTC policies, which are often still treated as marginal and sectoral, for the future sustainability of integrated care strategies.

**Supplementary Information:**

The online version contains supplementary material available at 10.1186/s12877-024-04687-x.

## Introduction

Population ageing and rising poverty are two of the most pressing issues today, even in Western European nations. By 2050, the over-60 population will double to 22% of the worldwide total, and the over-80 population will triple to 426 million, thus impacting the global need for long-term care (LTC). Moreover, population ageing often can be associated to situation of disabilities in the basic activities of daily living (ADL disabilities), which usually requires support in terms of Long-Term Care (LTC) for coping with the needs caused by ADL. When such support is not supplied (totally or partially) by the public social and health system, the consequent expenditures that families must sustain can increase the economic stress that families must face [1–2]. In parallel, the risk of poverty is also growing as a result of the recent international economic crisis and the COVID-19 containment measures, which have reduced individual and collective productivity and had a negative impact on household income [3–5]. In 2020, 21.5% of the European population was at risk of poverty or social exclusion [6]. Global health and welfare systems are strongly affected by these growing needs that threaten their sustainability [7–10]; therefore, reducing inequalities in health and social provision is essential for sustainable development in many countries [11]. In this regard, a recent study demonstrates that, in many countries, policies supporting informal care are seldom implemented to counteract the negative socio-economic impact on those who provide unpaid care, as these policies often consist of basic cash benefits or allowances that do not consider the real implications and costs of informal care [12]. Investigating the association between LTC needs and the risk of households’ socio-economic deprivation and risk of poverty is, therefore, a fundamental tool to better understand the complexity of the LTC challenge and improve support policies for dependent people and their caregivers in Europe. Recent literature has devoted a growing amount of attention to this topic; however, it has done so by focusing mostly on single-country studies analysing specific facets of this association. Woo and colleagues [13], for instance, examined the effects of older people’s health conditions on their income, while [14] investigated the impact of care expenditure on daily out-of-pocket (OOP) expenditures. Other studies have identified the inequities resulting as a consequence of the financial burden imposed by OOP health expenditures [15–16].

This study explores the relationship between LTC needs and the risk of poverty at the household level in eight European countries: Italy, Spain, Romania, Poland, Netherlands, Finland, Austria and Germany. Literature underlines how different care regimes and welfare models characterize these countries [17–19]. In 2014, Schulmann and Leichsenring [20] discussed the existing care models, underlying differences and similarities related to different classification characteristics used by the following Literature. Considering the eight selected countries, Lamura [17] and Nies [18] identified four care regimes in Europe based on typologies of care provision levels: (a) the familistic regime in Italy and Spain, characterized by a high demand for care, low formal care provision, and high informal care; (b) the standard care mix (Austria and Germany), where the medium/high demand for care is covered by a medium level of both informal and formal care provision; (c) the Universal-Nordic regime (Finland and the Netherlands) based on high formal care and low informal care provision to meet a medium level of care demand; and (d) the in transition regime (Poland and Romania), characterized by high informal care and medium formal care provision specifically aimed to cover a low level of care demand. This classification finds a partial similarity with the clustering proposed by Kraus et al. in 2010 [19], valorising the spending and the use of public or private resources, which defines: (a) Germany as an informal care-oriented care regime with low private spending; (b) the Netherlands, a generous, accessible and formalised care regime; (c) Austria, Finland and Spain informal care oriented with at high private financing; (d) Italy, Poland and Romania countries with high private financing, informal care seems a necessity. The two different classifications underline how the eight selected countries cover all different strategies existing in Europe, and the possibility of considering area aggregations (North-Western, Central Europe, Southern Europe and Eastern Europe) as an effective classification summarises them.

**Fig. 1:**
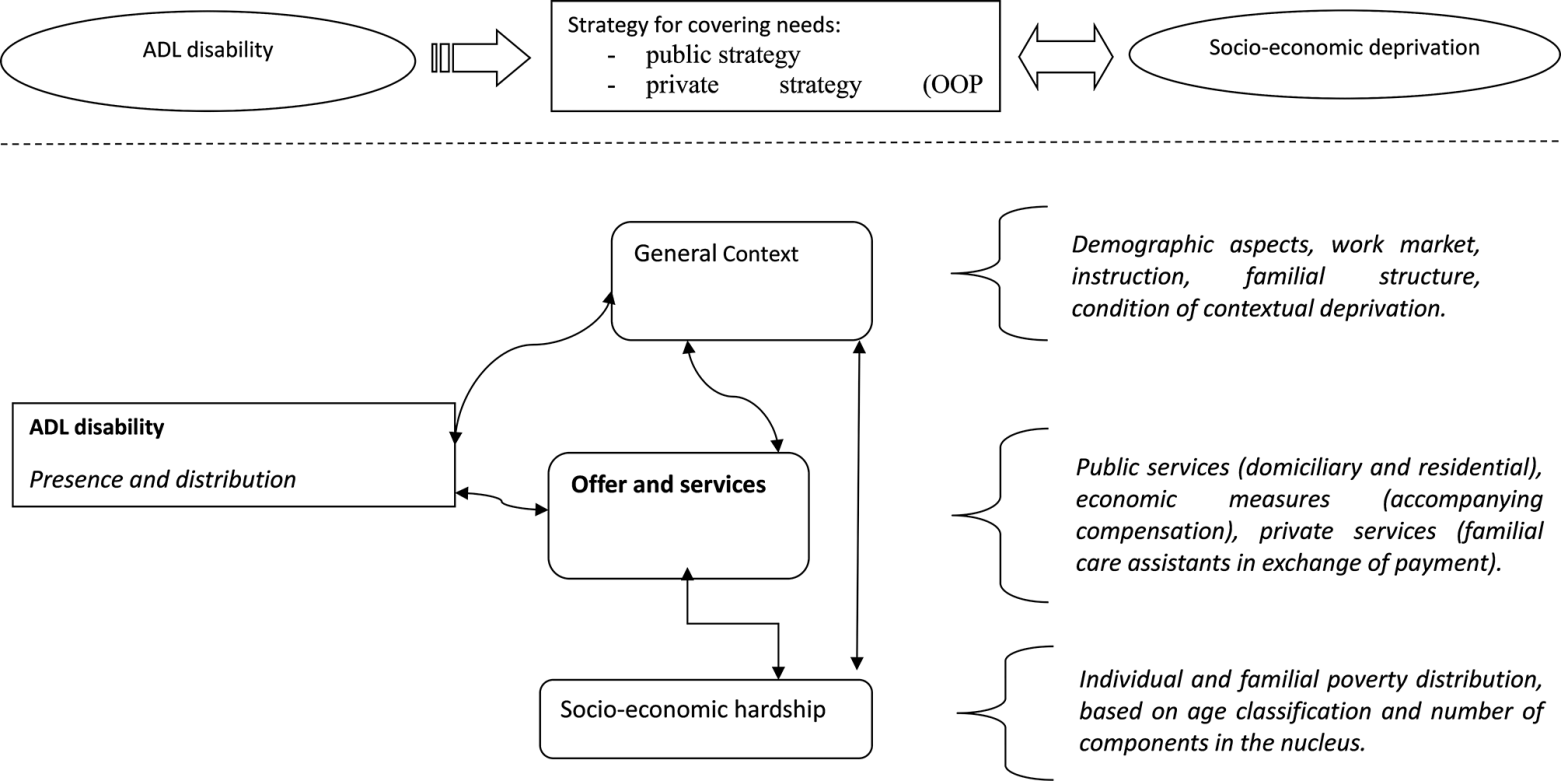
Conceptual framework: social and health needs, care system and socio-economic deprivation: the structure

The conceptual framework at the start of our study is reported in Fig. 1.

The selection of these countries is also based on their different positioning in terms of socio-economic conditions and LTC needs. In Romania, an estimated 34.4% of the population will be at risk of poverty and social exclusion in 2022, compared to about 15.9% in Poland, 16.3% in Finland, 17.5% in Austria and 16.5% in the Netherlands. In Germany, 20.9% of the population falls into this category, whereas in Italy and Spain this stands at 24.4% and 26.0%, respectively [6]. There are also substantial cross-national differences in terms of LTC needs: in Poland and Romania, more than 20% of those aged 65 + are estimated to be dependent; in the Netherlands and Spain, this category reaches 14.5% and 13.2%, respectively; in Austria and Italy, this stands at 16.3%; and in Germany, the proportion of 65 + dependent older people is 18.5% [21].

This paper advances the quantitative component of the project “Socio-Economic deprivation related to the effect of the presence of dependent older people: strategies for Innovative Policies in Europe” (SEreDIPE). The quantitative analysis attempts to identify the statistical correlation between ADL limitations in older people and the risk of poverty in order to evaluate the effects on households, also describing the main factors influencing the increasing or decreasing risk of poverty in Europe and across different care regimes.

## Materials and methods

Table 1 details the variables taken into consideration following exhaustive searches in the main international databases. These databases included the following:

**Table 1:** Variables collected for the study, by concept area, measurement level (absolute level – a.v., %) and source database

Area	Indicators	Consulted Database
Home care	Pop 65 + treated in integrated home care (%)	WHO
Residential care	Elderly care health facilities rate (%)	WHO
	Residential beds in nursing homes for the elderly (%)	HFA-Europe
	Residential beds in health and social residence for the elderly (%)	Eurostat
	Care workers for the elderly, by type of structure (%) (available only as total in structures)	WHO
Health Expenditure	Current public health expenditure per capita (%)	HFA-Europe
	Public health expenditure corresponded per capita in total convention for social benefits (% of GdP)	Eurostat
	Total Health Expenditure (THE), expressed in US$ purchasing power parity (ppp) per capita (a.v., % of GDP)	HFA-Europe
	Total government expenditure as % of GDP	HFA-Europe
	Public-sector health expenditure as % of total health expenditure & GDP	HFA-Europe
	Private-sector expenditure on health as % of total health expenditure & GDP	HFA-Europe
	Gross domestic product (GDP), expressed in US$ purchasing power parity (ppp) per capita	HFA-Europe
Coverage of services	Index of territorial coverage of services (per 100 pop.)	WHO
Cash benefits	% of total disability pensions on total population	Eurostat
	Average monthly amount for total disability pensions	Eurostat
	Average monthly amount of accompanying allowance for total invalids	Eurostat
Private services	Out-of-Pocket expenditure for health services (US$ppp per-capita)	HFA-Europe
	Out-of-Pocket expenditure for social services (US$ppp per-capita)	HFA-Europe
	Number of family assistants (carers) (per 100,000 population)	HFA-Europe
Population	Resident population by sex and age (%)	HFA-Europe
	Dependency ratio (%)	Computed by data from HFA-Europe
	Ageing index	HFA-Europe
Family	Average number of components	Eurostat
	Frequency of the number of components (from 1 to 6 member) (%)	Eurostat
	Older people (65 + years old) living alone (%)	Eurostat
Education	Literacy rate in population aged 15 + year	HFA-Europe
	% of population with postsecondary education aged 25 + year	HFA-Europe
	% of population with primary education only aged 25 + years	HFA-Europe
	% of population with secondary education only aged 25 + years	HFA-Europe
	Human Development Index	HFA-Europe
Employement	Active population rate (15–64) (%)	Eurostat
	Labour force (%)	HFA-Europe
	Unemployment rate (%)	HFA-Europe
	Youth unemployment rate (15–24) (%)	HFA-Europe
	Frequency of employment in economic sectors (Industry, Agriculture, Tertiary Sector and other activities) (%)	World Bank - World Development DB
Poverty	People at risk of poverty and social exclusion (%)	HFA-Europe
	Poor families (%)	Eurostat
	Incidence of poverty (people) (%)	Computed by data from Eurostat
	Frequency of poor families for no. of family members (1–6) (%)	Eurostat
	Poor families with at least 1 child (%)	Eurostat
	Poor families according to the structure (single-parent; with at least one child) (%)	Eurostat
	Distribution of poor couples by n. of children (1–3 +)	Eurostat
	Severe material deprivation by age (0–64, 65+) (%)	Eurostat
	Severe material deprivation by employment status (age 18+) (%)	Eurostat
	Severe material deprivation by education level (age 18+) (%)	Eurostat
Disability and care recipients	Disability rate (%)	HFA-Europe - Eurostat
	Disability rate by age group (6–64; 65+) (%)	HFA-Europe - Eurostat
	Disability rate in activities of daily living (ADL) (%)	HFA-Europe - Eurostat
	Older people with ADL limitations (%)	HFA-Europe - Eurostat
Life expectancy	Life expectancy in good health (yrs.)	HFA-Europe - Eurostat
	Expected healthy life years at age 65 (yrs.)	HFA-Europe

- Eurostat DB (https://ec.europa.eu/eurostat/data/database);
- Health for All Europe DB (https://gateway.euro.who.int/en/datasets/european-health-for-all-database/);
- WHO DB (https://www.who.int/data/collections);
- World Development DB from World Bank (https://data.worldbank.org/).

The stated variables were chosen according to the conceptual framework underpinning a pilot quantitative study previously conducted in Italy, which focused on the same topics and utilized comparable methodologies and statistical techniques [22]. This study investigated whether and how the identified variables tested for the presence of a correlation between the incidence of poverty and the presence of ADL disabilities, and defined the role of the applied public and private interventions to address the needs and characteristics of the population at national level.

The database was consulted for the period between 1990 and January 21, 2022 (most recent date for which information was available). The starting year was chosen because it was the first year after the fall of the Berlin Wall, when data across Europe became available and comparable.

All the variables found between 1990 and the most recent accessible year were taken into consideration. For the analyses, only those variables expressed as a percentage, rate, or in index form were taken into consideration in order to ensure the comparability of data across nations and years [22]. A preliminary standardization was applied in order to allow their comparability and usage in more complex models. In this way, it was possible to appreciate how they all showed a sufficiently regular distribution for each country, without the presence of outliers that could compromise their use. Each variable’s process of standardization used a historical trend of standard deviation as the normalizing element of the weighted average, considering the values observed year by year. In the next steps of the analysis, such standardized mean values (representing the total of the variable trend) were used.

The series of variables were checked for potential outliers. As none were found, all the variables in the analyses represent the average of each individual variable’s series.

In the final dataset for analysis, 104 variables were evaluated along with the classification by country (Austria, Finland, Germany, Italy, the Netherlands, Poland, Romania, and Spain).

These variables, which pertained to the following conceptual areas, were grouped as follows: home care, residential care, health expenditure, service coverage, cash benefits, private services, population, family, education, employment, poverty, disability and care recipients, and life expectancy.

In the following analyses, the dependent variable was the Incidence of Household poverty computed as the % of households who are said to be living in poverty if their income and resources are so inadequate as to preclude them from having a standard of living considered acceptable in the society in which they live (according to the 2004 Eurostat definition).

The definitions coming from the original database for the non-self-explicative variables were reported in the Supplementary Table S1.

The statistical analyses were conducted as described hereafter [22–23].

An analysis of the Pearson’s Bivariate Correlation between the dependent variable and all other variables in order to identify only those variables that statistically significantly correlated to the Poverty Index (statistical significance threshold at *p* < 0.05). This step was designed to reduce the number of covariates to be incorporated into the multivariate linear regression model.

A Multivariable Linear Regression Model was tested, in which the Poverty Index was the dependent variable and the covariates identified in the preceding phase were the independent variables. Various checks were performed during this analysis to exclude collinearity bias and unreliable results:

- Check of the adj. R^2^ of the model with statistical significance at *p* < 0.05;
- Consequently, the model was accepted at *p* < 0.05;
- Tolerance check of the variables for collinearity at *p* < 0.001.

Such analysis was replicated for the period before the pandemic insurgence (till to 2019), in order to evaluate possible effects due to the Covid-19 pandemic with respect to the previous period. The results were reported separately in Table S3 and briefly discussed in the “Results” paragraph.

Geographical clustering effects were checked by considering the potential similitude of the included countries across all the variables incorporated into in the study. The geographical clustering was aimed to stress possible similarities between countries and regimes, aggregating them if and where suggested by the analysis.

A reduced Multivariable Linear Regression Model for each European cluster was applied to the identified geographical clusters in order to analyze more specific aspects of the interactions between statistically significant covariates and the Poverty Index. All checks at point 2 were also conducted on these models.

It is important to stress that by these analyses we aimed to identify factors that that do not necessarily imply that could influence the changes in the Index of Household Poverty, without going deeper in analysing the causality if the relationship, which will be the aim of a next study.

The software packages SPSS 19.0 and STATA 14.0 were used to develop the analyses.

## Results

Table 2 illustrates the Pearson’s Bivariate Correlation Results, grouping variables into two groups: those associated with a reduction in the Poverty Index and those resulting in an increase.

**Table 2:** Variables correlated to the Poverty Index

Effects of reduction of the Poverty Index	Pearson’s r	Effects of increasing of the Poverty Index	Pearson’s r
Literacy rate in Females (F) aged 15 + year	-0.821	Three-persons families (%)	0.880
Literacy rate in population aged 15 + years	-0.778	Four-persons families (%)	0.841
Human Development Index	-0.842	People at risk of poverty and social exclusion (%) - F	0.927
One-person families (%)	-0.881	People at risk of poverty and social exclusion (%) - M	0.924
Two-persons families (%)	-0.784	People at risk of poverty and social exclusion (%) - All	0.927
GDP US$ppp per capita	-0.878	Three-persons poor households (%)	0.915
Total Health Expenditure -% of GDP	-0.745	Poor couples with at least one children (%)	0.803
Total Government Expenditure - % of the GDP	-0.737	Severe material deprived − 0–64 (%)	0.814
Public Health Expenditure - % of the GDP	-0.738	Severe material deprived − 65+ (%)	0.782
Attendance allowance by person (ppp, monthly avg.)	-0.961	Severe material deprived - Employed (%)	0.793
Residential beds in nursing home for the elderly (per-100,000-population)	-0.924	Severe material deprived - Not employed (%)	0.795
Index of territorial coverage of the services (per 100 pop.)	-0.729	Severe material deprived - Retired (%)	0.754
Public health expenditure corresponded per capita in total convention for social benefits (% of GDP)	-0.834	Severe material deprived - Others outside labour force (%)	0.754
Elderly care health facilities rate (% on 65 + pop.)	-0.860	Severe material deprived - Till to lower secondary education level (%)	0.769
Pop 65 + treated in integrated home care (%)	-0.795	Severe material deprived - Upper secondary education level (%)	0.774
Care workers for the elderly in residential care (%)	-0.715	Severe material deprived - Tertiary education level (%)	0.817
		Disability Rate − 65 + All (% on 65 + All)	0.887
		Disability Rate − 65 + M (% on 65 + M)	0.845
		Disability Rate − 65 + F (% on 65 + F)	0.900
		Private OOP household health expenditure - % of THE	0.666

N.B.: Only variables correlated with statistical significance at *p* < 0.05

The results indicate that a good level of education, a small family size, and, not surprisingly, a high income is associated with a reduction in household poverty. However, aspects of public investments in health and social support (e.g., public health expenditure per capita dedicated to social benefits, index of the service’s territorial coverage, etc.) work to contrast the factors which contribute to increase poverty.

In contrast, factors such as the presence of a disability, private out-of-pocket (OOP) health expenditures, and the previous presence of material poverty, among others, were found to exacerbate household deprivation.

As expected, cancer prevalence was also significantly correlated to the Poverty Index, resulting in its increase in the six countries for which this data was available.

The Multivariable Linear Regression Model was applied to all eight countries to evaluate which variables were co-responsible for the primary effects in reducing or increasing household poverty. The results are presented in Table 3.

**Table 3:** Results of the multivariable linear regression model applied to the eight countries

	Unstandardized Coefficients B	*P* < 0.05
Literacy rate in population aged 15 + years	-5.298	0.000
One-person families (%)	-0.099	0.000
Index of territorial coverage of the service (per 100 pop.)	-0.031	0.000
Population aged 65 + years treated in integrated home care (%)	-0.500	0.000
Care workers for the elderly in residential care (%)	-5.480	0.000
Poor couples with at least one children (%)	0.470	0.000
Private OOP household health expenditure (% of Total Health Expenditure)	0.458	0.000

Dependent variable: Incidence of Household Poverty; **adj. R2** = 0.988

As the above results demonstrate, the model is statistically significant since the variables displayed were statistically significant, passed the tolerance check for collinearity, and were common to all eight countries.

The variables that are negatively correlated to the risk of poverty pertained to the area of policy intervention and service provision: the index of the service’s territorial coverage; the proportion of individuals aged 65 + receiving integrated home care; and the number of care workers in residential care facilities for the elderly. This latter factor, coupled with the personal traits of a good level of education, was the most effective way to counteract a rise in household poverty.

On the opposite side, increasing private OOP household health expenditures and the existence of “poor” couples with at least one child are two factors that are positively correlated to a rise in poverty.

It is noteworthy that, considering the data coming from the period before the pandemic insurgence, there were not sensitive differences in the results coming from this model (see Table S3 in the Supplementary Tables).

Table 4 presents the results of the analysis on possible geographical clustering effects.

**Table 4:** Evaluation of the presence of geographic clusters

European Clusters	Odd Ratio (OR)	Significance
North-Western and Central Europe	1	
Southern Europe	8.12	0.004
Eastern Europe	9.70	0.002

Dependent variable: Incidence of Household Poverty; **adj. R2** = 0.873

*Note*: *North-Western and Central Europe = Austria, Finland, Germany and the Netherlands; Southern Europe = Italy, Spain; Eastern Europe = Poland, Romania*

The countries were clustered into three groups based on their greatest affinity in terms of the variables under consideration: North-Western and Central Europe (Austria, Finland, Germany, and the Netherlands); Southern Europe (Italy and Spain); and Eastern Europe (Poland and Romania). The statistically significant odds ratio (OR) associated with the clusters confirmed the presence of a geographical clustering effect as a result of the countries’ correspondence to the European macro-area. These findings also revealed how care regimes are aggregated according to the countries’ socio-economic characteristics. The North-Western and Central European cluster comprised four nations with similar high-level socio-economic conditions and two distinct care regimes: the mixed-care regime and the Universal-Nordic regime. The Southern European cluster gathered two countries with familistic care regimes, whereas the Eastern European cluster grouped countries with care regimes in transition.

This result suggests the presence of other variables characterizing the geographical groups, in addition to the variables already specified. Therefore, regression procedures were also computed for each cluster to validate this possibility. The results are presented in Table 5.

**Table 5:** Further statistically significant variables influencing the Poverty Risk, by European clusters (only statistically significant variables not reported in Table 3)

European Clusters	Unstandardized Coefficients B	Sig.
*North-Western and Central Europe*		
Four-persons families (%)	0.050	0.000
Severe material deprived - Tertiary Education Level (%)	2.414	0.000
Residential beds in nursing home for the elderly (per 100,000)	-0.404	0.000
*Southern Europe*		
Residential beds in nursing home for the elderly (per 100,000)	-0.101	0.000
*Eastern Europe*		
Residential beds in nursing home for the elderly (per 100,000)	-0.045	0.000

Dependent variable: Incidence of Household Poverty. All the three linear regression models were statistically significant (*p* < 0.05)

A higher presence of residential beds in nursing homes for the elderly was a variable common to the three clusters capable of counteracting the incidence of poverty (when considered separately). In North-Western and Central European countries only, four-person families (%) and severe material deprivation with a tertiary level of education (%) were two additional variables which could contribute to an increase in the risk of poverty.

## Discussion

This study’s findings highlight the existence of a clear association between the need for LTC and the risk of poverty in households in Europe. The rapid ageing of the population and the resulting increase in the need for LTC compel experts and stakeholders to view this issue as an emerging key challenge for national and international health, social, and welfare systems. These results particularly underline the central role of LTC policies, which are often still treated as marginal and sectoral, for the future sustainability of integrated care strategies [24–25].

In this regard, it should be noted that, in recent decades, many European countries have implemented a progressive and partial decentralisation and privatisation of the LTC sector, shifting the responsibility for financing LTC services from the societal to the individual level [26–29]. A thorough examination of the factors associated with the risk of household poverty, as highlighted by this study, might provide some useful suggestions for the development of a sustainable strategy in this regard.

A first indication that emerges from the findings is that higher private spending on health care is associated with an increased risk of poverty for households, while higher public investments in the LTC sector decrease the risk of poverty for households. Therefore, efforts made by the government to improve and strengthen LTC services and interventions provide a clear safeguard for the economic sustainability of families. In particular, larger families face a greater risk of impoverishment than smaller ones in the event of LTC needs, highlighting one of the dimensions of inequities that the LTC risk imposes on the population. The literature suggests that this is potentially related to a decline in the ability of European families to provide informal care in a stable socio-economic environment. Informal caregivers are indeed more likely to face social exclusion marked by low life and/or income satisfaction due to their diminished potential to acquire gainful employment on the labour market, on the one hand, and isolation as a result of the high number of hours devoted to care, on the other [30–32]. In Mediterranean or Eastern European countries where the family is the primary provider of assistance, the risk of impoverishment is heightened because the economic and social support provided by families cannot fully compensate for the traditional lack of public service provision [22; 33–34].

Another key result that emerged from this study is that living in already disadvantaged conditions increases the probability of sliding into poverty in the presence of LTC needs [35–36]. The characteristics of the geographical clusters (or macro-areas) corroborate these findings, highlighting the importance of the household’s socio-economic conditions over and beyond the differences between the various care regimes. The few discrepancies between the three European macro-area clusters underline the central role of public investments in the provision of LTC services as a crucial tool to counteract the socio-economic disadvantages resulting from the escalation of LTC needs within the household. In this respect, the literature considers residential care beds as a proxy variable for the quality of the public offer of LTC services in Europe [8; 37].

The above results may contribute to the debate on the “right” mix of different types of LTC care–formal/informal, in-kind/cash, home/residential–within the forthcoming European LTC strategy, which is expected to be launched in 2022. This is also crucial in light of current demographic trends, which indicate an increase in the number of older Europeans living alone or in smaller households [6], with a consequent reduction in the potential number of informal carers, necessitating innovative LTC policies that go beyond the current ageing-in-place options.

However, it is important to recognize the limitations of this study. First of all, the comparative study based on national secondary data provides a framework for the analysis of the relationship, but does not allow for the detection of intra-national, regional, and local differences that exist in many European countries (e.g., Italy, Spain, and Germany). Secondly, the study does not include the effects of the recent COVID-19 crisis due to the unavailability of updated data regarding the pandemic’s impact, nor those following the outbreak of the Russian-Ukrainian war. We don’t know if such aspects have a relevant impact on the results, but a such long time series of data for variables describing the structural characteristics of national systems is unlikely to be influenced significantly by such conjunctural effects (particularly the pandemic crisis which had a shorter lasting than the war) with respect to the aims that our study pursued. However, these aspects might be the topic of a next study, aimed to going deeper on the changes. Thirdly, the use of a variable dependent on the risk of poverty does not allow for an evaluation of the aspects of social deprivation that informal carers typically experience.

## Conclusion

Even if the study was not aimed to find a causal relationship, the results suggests that the public provision of adequate LTC services appears to be an adequate strategy for mitigating the risk of household poverty occurring as a consequence of LTC needs. Policymakers could use the suggestion coming from the factors that our study has been found to be correlated with changes in household poverty as driving elements to advance innovative LTC policies and reduce the risk of material deprivation for dependent older families.

Despite the cited limitations, this study provides an innovative analysis of the relationship between the presence of LTC needs and the risk of household poverty. In this regard, future studies could certainly benefit from investigating related topics that could not be addressed by the present study, such as a comparative analysis of geographical differences conducted at local or non-national levels (e.g., NUTS regions), a specific study of the effects of the COVID-19 pandemic (considering different variables pertaining to the Health and LTC systems) and/or of the war in the Ukraine on the analyzed relationship, and, last but not least, the role of specific LTC needs that have a particular impact on the quality of life of family carers (e.g., dementia).

Considering the general value and hints coming from our results, the next steps for future studies should be devoted to explore the situation at regional/local level in any single country, trying to stress differences and suggest more specific solutions that, at the present ecological level cannot be identified.

### Electronic supplementary material

Below is the link to the electronic supplementary material.

**Supplementary Material 1: Table S1.** Definition of the non-self-explicative variables collected for the study, by concept area. **Table S2.** Average value for all variables for each country. **Table S3.** Results of the multivariable linear regression model applied to the eight countries

## Data Availability

The datasets generated during and/or analysed during the current study are not publicly available because of direct results of authors’ work, but are available from the corresponding author on reasonable request. Moreover, the public dataset that provide data from which the generated datasets originated, are available at: Eurostat DB (https://ec.europa.eu/eurostat/data/database); Health for All Europe DB (https://gateway.euro.who.int/en/datasets/european-health-for-all-database/); WHO DB (https://www.who.int/data/collections); World Development DB from World Bank (https://data.worldbank.org/).
