# Supplementary material for: Long-term care needs and the risk of household poverty across Europe: a comparative secondary data study
Source: BMC Geriatr. 2024 Jan 26;24:101. doi: 10.1186/s12877-024-04687-x (PMC10811889; doi:10.1186/s12877-024-04687-x)
Supplement: Supplementary file 1 — Supplementary Material 1: Table S1. Definition of the non-self-explicative variables collected for the study, by concept area. Table S2. Average value for all variables for each country. Table S3. Results of the multivariable linear regression model applied to the eight countries [file 12877_2024_4687_MOESM1_ESM.docx]

Supplementary Table S1. Definition of the non-self-explicative variables collected for the study, by concept area

| **Area** | **Indicators** | **Definition** |  |
| --- | --- | --- | --- |
| Health Expenditure | Public health expenditure corresponded per capita in total convention for social benefits (% of GdP) | The expenditure for social benefits/protection which completely covered by public funds. |  |
| Coverage of services | Index of territorial coverage of services (per 100 pop.) | The index combines 14 tracer indicators of service coverage into a single summary measure. The tracer indicators are as follows, organized by four components of service coverage: 1. Reproductive, maternal, newborn and child health 2. Infectious diseases 3. Noncommunicable diseases 4. Service capacity and access. |  |
| Cash benefits | % of total disability pensions on total population | Disability pensions are granted simply for having a permanent disability. |  |
|  | Average monthly amount for total disability pensions |  |  |
|  | Average monthly amount of accompanying allowance for total invalids |  |  |
| Population | Resident population by sex and age (%) | The considered age groups were: 0-14; 15-64; 65+. |  |
|  | Ageing index (%) | The % of elderly population (aged 65 years and over) on individuals younger than 14 years old in a specific population. |  |
| Poverty | People at risk of poverty and social exclusion (%) | At risk of poverty or social exclusion corresponds to the % of persons who are either at risk of poverty, or severely materially and socially deprived or living in a household with a very low work intensity with respect to the total population. People are included only once even if they are in more than one of these situations. |  |
|  | Severe material deprivation by age (0-64, 65+) (%) | The enforced inability to pay for at least four of the following nine items: 1) to pay their rent, mortgage or utility bills; 2) to keep their home adequately warm; 3) to face unexpected expenses; 4) to eat meat or proteins regularly; 5) to go on holiday; 6) a television set; 7) a washing machine; 8) a car; 9) a telephone. |  |
|  | Severe material deprivation by employment status (age 18+) (%) |  |  |
|  | Severe material deprivation by education level (age 18+) (%) |  |  |
| Disability and care recipients | Disability rate (%) | % of people with limitation in activities people usually do because of health problems for at least the past six months. |  |
|  | Disability rate by age group (6-64; 65+) (%) |  |  |
|  | Disability rate in activities of daily living (ADL) (%) |  |  |
|  | Older people with ADL limitations (%) |  |  |

Supplementary Table S2. Average value for all variables for each country

|  | **AUT** | **DEU** | **ESP** | **FIN** | **ITA** | **NLD** | **POL** | **ROU** | ***Totale*** |
| --- | --- | --- | --- | --- | --- | --- | --- | --- | --- |
| Dependency Ratio (%) - M | 43.5 | 43.1 | 43.8 | 45.5 | 45.5 | 45.1 | 42.3 | 43.6 | *44.0* |
| Dependency Ratio (%) - F | 53.0 | 54.3 | 50.5 | 55.5 | 53.3 | 51.5 | 49.2 | 49.4 | *52.1* |
| Dependency Ratio (%) - All | 48.2 | 48.6 | 47.1 | 50.5 | 49.4 | 48.3 | 45.8 | 46.5 | *48.1* |
| Ageing Index - F | 19.3 | 21.1 | 18.6 | 18.8 | 20.9 | 16.5 | 15.1 | 15.8 | *18.2* |
| Ageing Index - M | 13.0 | 14.5 | 14.0 | 12.8 | 14.5 | 13.3 | 9.8 | 11.5 | *12.9* |
| Ageing Index - All | 16.1 | 17.8 | 16.3 | 15.8 | 17.7 | 14.9 | 12.5 | 13.6 | *15.6* |
| Literacy rate in F aged 15+ year | 99.0 | 99.0 | 96.9 | 99.6 | 97.9 | 99.0 | 99.3 | 96.8 | *98.4* |
| Literacy rate in M aged 15+ year | 99.0 | 99.0 | 98.4 | 99.6 | 98.6 | 99.0 | 99.4 | 98.6 | *99.0* |
| Literacy rate in population aged 15+ yea | 99.0 | 99.0 | 97.7 | 99.6 | 98.3 | 99.0 | 99.3 | 97.7 | *98.7* |
| % of population with postsecondary education aged 25+ year | 24.8 | 31.5 | 25.0 | 24.6 | 12.5 | 28.9 | 22.1 | 15.6 | *23.1* |
| % of population with primary education only aged 25+ years | 1.1 | 3.2 | 20.0 | 4.7 | 20.9 | 9.8 | 17.6 | 10.6 | *11.0* |
| % of population with secondary education only aged 25+ years | 73.1 | 66.3 | 42.3 | 60.5 | 60.2 | 59.7 | 57.4 | 71.3 | *61.3* |
| Human Development Index | .8 | .9 | .8 | .9 | .8 | .9 | .8 | .7 | *.8* |
| Active Population - F% | 65.4 | 64.8 | 66.4 | 64.3 | 65.2 | 66.0 | 67.0 | 67.0 | *65.8* |
| Active Population - M% | 69.7 | 69.9 | 69.5 | 68.7 | 68.7 | 68.9 | 70.3 | 69.6 | *69.4* |
| Active Population - All% | 67.5 | 67.3 | 68.0 | 66.5 | 66.9 | 67.4 | 68.6 | 68.3 | *67.6* |
| Labour Force - All% | 53.9 | 53.6 | 45.7 | 53.2 | 43.0 | 57.5 | 49.3 | 52.0 | *51.0* |
| Unemployment rate - All | 4.5 | 8.2 | 17.2 | 9.5 | 9.9 | 5.2 | 12.4 | 6.9 | *9.2* |
| Youth unemployment rate (15-24) (%) - F | 7.9 | 8.1 | 38.1 | 24.1 | 34.4 | 8.7 | 28.4 | 21.0 | *21.3* |
| Youth unemployment rate (15-24) (%) - M | 8.3 | 9.9 | 32.8 | 25.3 | 27.9 | 8.7 | 25.1 | 20.2 | *19.8* |
| Youth unemployment rate (15-24) (%) - All | 8.1 | 9.0 | 35.4 | 24.7 | 31.1 | 8.7 | 26.8 | 20.6 | *20.6* |
| Agriculture employment (%) | 4.5 | 1.5 | 4.4 | 4.2 | 3.9 | 2.4 | 11.9 | 27.7 | *7.6* |
| Industry employment (%) | 26.1 | 28.3 | 21.3 | 22.8 | 27.2 | 17.0 | 31.1 | 28.8 | *25.3* |
| Services employment (%) | 69.4 | 70.2 | 74.3 | 72.9 | 68.9 | 80.5 | 57.0 | 43.5 | *67.1* |
| Average number of family components | 2.3 | 2.0 | 2.6 | 2.1 | 2.4 | 2.2 | 2.8 | 2.7 | *2.4* |
| Older people (65+ years old) living alone (%) | 13.1 | 13.7 | 10.6 | 14.9 | 14.9 | 11.6 | 12.9 | 15.1 | *13.3* |
| One-person families (%) | 35.9 | 40.0 | 23.7 | 40.6 | 30.6 | 36.7 | 24.3 | 26.7 | *32.3* |
| Two-persons families (%) | 29.2 | 34.2 | 29.3 | 34.3 | 27.3 | 32.3 | 24.8 | 26.2 | *29.7* |
| Three-persons families (%) | 15.6 | 12.7 | 21.2 | 10.9 | 20.1 | 12.2 | 20.0 | 18.7 | *16.4* |
| Four-persons families (%) | 12.5 | 9.6 | 18.5 | 9.3 | 16.8 | 13.0 | 16.5 | 16.5 | *14.1* |
| Five-persons families (%) | 4.7 | 2.6 | 5.3 | 3.5 | 4.1 | 4.4 | 7.9 | 7.0 | *4.9* |
| Six-and_more persons families | 2.0 | .9 | 2.0 | 1.5 | 1.1 | 1.4 | 6.4 | 4.9 | *2.5* |
| People at risk of poverty and social exclusion (%) - F | 19.8 | 21.3 | 26.3 | 17.9 | 28.5 | 16.6 | 31.5 | 43.0 | *25.6* |
| People at risk of poverty and social exclusion (%) - M | 16.8 | 18.5 | 24.9 | 16.3 | 24.8 | 14.7 | 30.4 | 41.3 | *23.5* |
| People at risk of poverty and social exclusion (%) - All | 18.3 | 19.9 | 25.6 | 17.1 | 26.7 | 15.7 | 31.0 | 42.2 | *24.6* |
| Poor households (%) | 12.7 | 12.3 | 13.8 | 13.5 | 12.2 | 10.6 | 12.3 | 14.4 | *12.7* |
| Incidence of poverty (%) - All persons | 16.7 | 17.6 | 25.1 | 15.2 | 23.3 | 14.7 | 23.4 | 28.1 | *20.5* |
| Three-persons poor households (%) | 9.3 | 9.5 | 12.3 | 9.9 | 10.7 | 7.3 | 11.4 | 12.8 | *10.4* |
| One-person poor households (%) | 13.3 | 17.6 | 14.1 | 21.0 | 12.8 | 17.7 | 13.2 | 15.9 | *15.7* |
| Two-persons poor households (%) | 16.1 | 18.2 | 16.4 | 16.6 | 13.6 | 18.2 | 15.7 | 15.8 | *16.3* |
| Poor families with at least 1 child (%) | 15.8 | 12.2 | 15.1 | 12.5 | 13.4 | 9.5 | 12.5 | 13.5 | *13.1* |
| Poor single-parent families (%) | 27.5 | 28.3 | 23.5 | 26.6 | 20.7 | 29.2 | 22.8 | 22.5 | *25.1* |
| Poor couples with at least one children (%) | 17.3 | 13.1 | 17.0 | 12.1 | 16.4 | 9.4 | 14.9 | 20.2 | *15.0* |
| Severe material deprived - 0-64 (%) | 4.1 | 5.1 | 5.7 | 3.2 | 9.6 | 2.6 | 13.0 | 25.7 | *8.6* |
| Severe material deprived - 65+ (%) | 2.0 | 2.7 | 2.8 | 1.8 | 8.0 | .8 | 14.2 | 27.2 | *7.4* |
| Severe material deprived - Employed (%) | 2.2 | 2.7 | 3.2 | 1.1 | 6.1 | 1.0 | 8.8 | 21.1 | *5.8* |
| Severe material deprived - Not employed (%) | 5.0 | 6.6 | 6.2 | 5.0 | 11.5 | 3.8 | 17.5 | 28.3 | *10.5* |
| Severe material deprived - Unemployed (%) | 19.8 | 26.8 | 13.7 | 14.3 | 24.4 | 16.6 | 29.1 | 48.2 | *24.1* |
| Severe material deprived - Retired (%) | 2.6 | 2.8 | 2.5 | 2.1 | 6.0 | .8 | 13.0 | 25.3 | *6.9* |
| Severe material deprived - Others outside labour force (%) | 5.4 | 6.8 | 5.1 | 6.4 | 12.4 | 3.7 | 18.6 | 31.3 | *11.2* |
| Severe material deprived - Till to lower secondary (%) | 7.2 | 9.0 | 6.4 | 4.4 | 12.7 | 3.4 | 23.0 | 37.9 | *13.0* |
| Severe material deprived - Upper secondary (%) | 2.9 | 4.1 | 4.1 | 3.4 | 6.2 | 2.2 | 12.9 | 19.9 | *7.0* |
| Severe material deprived - Tertiary (%) | 1.3 | 2.1 | 1.8 | .9 | 2.7 | 1.1 | 3.3 | 6.1 | *2.4* |
| Life expectancy at birth in good health - M | 72.6 | 72.8 | 73.8 | 72.8 | 73.8 | 76.2 | 64.2 | 66.9 | *71.6* |
| Life expectancy at birth in good health - F | 76.2 | 76.2 | 76.7 | 78.0 | 76.1 | 78.8 | 68.9 | 71.4 | *75.3* |
| Life expectancy at birth in good health - All | 74.5 | 74.6 | 75.2 | 75.4 | 75.0 | 77.6 | 66.5 | 69.1 | *73.5* |
| Disability Rate - 15-64 All (% on 15-64 All) | 11.5 | 16.9 | 11.3 | 14.6 | 6.1 | 16.2 | 11.7 | 10.3 | *12.3* |
| Disability Rate - 15-64 M (% on 15-64 M) | 11.7 | 16.2 | 9.7 | 14.3 | 5.2 | 13.2 | 10.8 | 9.4 | *11.3* |
| Disability Rate - 15-64 F (% on 15-64 F) | 11.4 | 17.6 | 12.9 | 14.9 | 7.0 | 19.1 | 12.5 | 11.1 | *13.3* |
| Disability Rate - 65+ All (% on 65+ All) | 35.3 | 34.2 | 37.9 | 25.8 | 40.7 | 21.4 | 49.0 | 52.3 | *37.1* |
| Disability Rate - 65+ M (% on 65+ M) | 30.3 | 30.5 | 29.7 | 23.8 | 34.4 | 17.3 | 37.0 | 47.6 | *31.3* |
| Disability Rate - 65+ F (% on 65+F) | 39.1 | 37.1 | 44.3 | 27.3 | 45.4 | 26.2 | 56.0 | 56.4 | *41.5* |
| Disability Rate - 15+ All (% on 15+ All) | 16.4 | 21.0 | 16.7 | 17.1 | 14.5 | 17.2 | 17.7 | 18.0 | *17.3* |
| Disability Rate - 15+ M (% on 15+ M) | 15.0 | 19.2 | 13.3 | 16.2 | 11.5 | 14.0 | 14.1 | 15.9 | *14.9* |
| Disability Rate - 15+ F (% on 15+ F) | 17.6 | 22.6 | 20.0 | 18.0 | 17.2 | 20.4 | 21.1 | 20.2 | *19.6* |
| Expected healthy life years after 65 - M | 8.0 | 7.9 | 10.0 | 8.2 | 8.5 | 10.2 | 7.4 | 6.4 | *8.3* |
| Expected healthy life years after 65 - F | 7.9 | 8.2 | 9.5 | 8.7 | 8.1 | 10.2 | 8.2 | 5.9 | *8.3* |
| Disability rate in activities of daily living (ADL) (%) - 55-64 F | 1.5 | 2.4 | .9 | .8 | 1.1 | 3.3 | 1.4 | 1.4 | *1.6* |
| Disability rates in activities of daily living (ADL) (%) - 65+ F | 13.1 | 6.7 | 12.5 | 4.8 | 12.9 | 10.0 | 9.7 | 10.2 | *10.0* |
| Disability rates in activities of daily living (ADL) (%) - 55+ F | 8.4 | 5.1 | 8.0 | 3.4 | 8.7 | 7.3 | 6.4 | 7.0 | *6.8* |
| Disability rates in activities of daily living (ADL) (%) - 55-64 M | 1.2 | 1.5 | 1.1 | 1.3 | .7 | 1.4 | 2.2 | 1.3 | *1.3* |
| Disability rates in activities of daily living (ADL) (%) - 65+ M | 6.9 | 3.9 | 5.0 | 1.9 | 6.5 | 4.7 | 6.7 | 7.6 | *5.4* |
| Disability rates in activities of daily living (ADL) (%) - 55+ M | 4.4 | 2.8 | 3.3 | 1.7 | 4.2 | 3.3 | 4.5 | 4.8 | *3.6* |
| Disability rates in activities of daily living (ADL) (%) - 55-64 All | 1.4 | 1.9 | 1.0 | 1.1 | .9 | 2.3 | 1.7 | 1.4 | *1.5* |
| Disability rates in activities of daily living (ADL) (%) - 65+ All | 10.3 | 5.5 | 9.2 | 3.4 | 10.1 | 7.5 | 8.5 | 9.2 | *8.0* |
| Disability rates in activities of daily living (ADL) (%) - 55+ All | 6.5 | 4.0 | 5.9 | 2.6 | 6.6 | 5.4 | 5.6 | 6.1 | *5.3* |
| GDP - USppp per capita | 36818.5 | 34659.5 | 22647.0 | 36120.2 | 28464.9 | 38474.6 | 8123.4 | 5221.4 | *26316.2* |
| Total Health Expenditure - USppp per capita | 3001.3 | 2972.4 | 1834.9 | 2209.2 | 2157.9 | 2901.2 | 730.3 |  | *2258.2* |
| Private OOP household health expenditure - % of THE | 16.1 | 12.6 | 22.2 | 20.3 | 22.8 | 7.1 | 26.0 | 20.1 | *18.4* |
| Total Health Expenditure -% of GDP | 10.5 | 10.5 | 8.2 | 8.3 | 8.4 | 9.0 | 6.2 | 4.9 | *8.2* |
| Total Government Expenditure - % of the GDP | 52.1 | 46.5 | 41.8 | 53.3 | 48.7 | 44.6 | 44.7 | 36.2 | *46.0* |
| Public Health Expenditure - % of THE | 74.8 | 77.0 | 73.2 | 74.5 | 76.1 | 70.5 | 72.3 | 79.6 | *74.7* |
| Private Health Expenditure - % of THE | 25.1 | 22.1 | 27.5 | 26.5 | 25.8 | 25.1 | 29.3 | 20.4 | *25.2* |
| Public Healt Expenditure - % of the GDP | 7.8 | 8.2 | 6.0 | 6.1 | 6.3 | 6.9 | 4.4 | 3.9 | *6.2* |
| Private Health Expenditure - % of the GDP | 2.6 | 2.3 | 2.3 | 2.2 | 2.2 | 2.1 | 1.8 | 1.0 | *2.1* |
| Disability pension by person (ppp, monthly avg.) | 374.9 | 30.5 | 275.2 | 477.4 | 144.4 | 697.1 | 162.4 | 50.6 | *276.6* |
| Attendance allowance by person (ppp, monthly avg.) | 621.6 | 662.4 | 358.4 | 875.7 | 354.9 | 814.2 | 232.3 | 146.9 | *508.3* |
| Residential beds in nursing home for the elderly (per-100000) | 726.4 | 947.7 | 467.8 | 864.1 | 308.4 | 1105.7 | 207.6 | 103.0 | *591.3* |
| Number of family assistants (carers) (per-100000) | 402.2 | 489.8 | 772.6 | 2304.0 | 889.0 | 689.3 | 302.7 | 279.7 | *766.2* |
| Index of territorial coverage of the service (per 100 pop.) | 69.8 | 67.5 | 64.5 | 66.0 | 68.3 | 68.3 | 65.3 | 56.3 | *65.7* |
| Public health expenditure corr. per capita in conv. tot. for social benefits (%) | 23.3 | 26.6 | 18.0 | 27.6 | 21.1 | 21.5 | 13.0 | 9.5 | *20.1* |
| Elderly care health facilities rate (% on 65+ pop.) | 4.3 | 4.2 | 2.2 | 4.3 | 3.8 | 4.5 | .8 | .6 | *3.1* |
| Pop 65+ treated in integrated home care (%) | 11.2 | 14.2 | 9.3 | 9.1 | 6.3 | 12.6 | 5.1 | 2.9 | *8.8* |
| Care workers for the elderly in structure (%) | 2.1 | 2.2 | 2.4 | 2.3 | 1.9 | 2.5 | 1.2 | .7 | *1.9* |
| Residential beds in health and social residence for the elderly (per-100000) | 47.8 | 151.0 | 53.4 | 238.1 | 18.3 | 42.2 | 13.8 | 117.4 | *85.3* |
| F% - 00-14 | 15.3 | 14.1 | 14.9 | 17.1 | 13.6 | 17.4 | 17.8 | 17.1 | 15.9 |
| F% - 15-64 | 65.4 | 64.8 | 66.4 | 64.3 | 65.2 | 66.0 | 67.0 | 67.0 | 65.8 |
| F% - 65+ | 19.3 | 21.1 | 18.7 | 18.7 | 21.1 | 16.6 | 15.2 | 16.0 | 18.3 |
| M% - 00-14 | 17.1 | 15.6 | 16.4 | 18.6 | 15.3 | 18.6 | 19.9 | 18.7 | 17.5 |
| M% - 15-64 | 69.7 | 69.9 | 69.5 | 68.7 | 68.7 | 68.9 | 70.3 | 69.6 | 69.4 |
| M% - 65+ | 13.2 | 14.5 | 14.1 | 12.7 | 16.0 | 12.5 | 9.8 | 11.6 | 13.0 |
| All% - 00-14 | 16.2 | 14.8 | 15.6 | 17.8 | 14.4 | 18.0 | 18.8 | 17.9 | 16.7 |
| ALL% - 15-64 | 67.5 | 67.3 | 68.0 | 66.5 | 66.9 | 67.4 | 68.6 | 68.3 | 67.6 |
| ALL% - 65+ | 16.3 | 17.9 | 16.4 | 15.7 | 18.6 | 14.6 | 12.6 | 13.8 | 15.7 |

Supplementary Table S3. Results of the multivariable linear regression model applied to the eight countries

|  | ***Unstandardized Coefficients B*** | ***P < 0.05*** |
| --- | --- | --- |
| Literacy rate in population aged 15+ years | -5.302 | 0.000 |
| One-person families (%) | -0.098 | 0.000 |
| Index of territorial coverage of the service (per 100 pop.) | -0.285 | 0.000 |
| Population aged 65+ years treated in integrated home care (%) | -0.501 | 0.000 |
| Care workers for the elderly in residential care (%) | -5.505 | 0.000 |
| Poor couples with at least one children (%) | 0.472 | 0.000 |
| Private OOP household health expenditure (% of Total Health Expenditure) | 0.458 | 0.000 |

**Dependent variable:** Incidence of Household Poverty; **adj. R2** = 0.988.
